# Safety and Efficacy of Direct Oral Anticoagulants Versus Warfarin Following WATCHMAN in High-Risk Patients

**DOI:** 10.1016/j.jscai.2022.100042

**Published:** 2022-04-20

**Authors:** Bryan E-Xin Tan, Mohan Rao, Bipul Baibhav, Dmitry Chuprun, Abrar Shah, Deepak L. Bhatt, Jeremiah P. Depta

**Affiliations:** a Department of Internal Medicine, Rochester General Hospital, Rochester, New York; b Sands-Constellation Heart Institute, Rochester Regional Health, Rochester, New York; c Brigham and Women’s Hospital Heart & Vascular Center, Harvard Medical School, Boston, Massachusetts

**Keywords:** WATCHMAN, left atrial appendage occlusion, novel oral anticoagulant, warfarin, vitamin K antagonist

## Abstract

**Background:**

In the pivotal WATCHMAN trials, warfarin was used exclusively for postprocedural anticoagulation following left atrial appendage closure. We sought to investigate the safety and efficacy of direct oral anticoagulants (DOACs) in high-risk patients with atrial fibrillation who underwent left atrial appendage closure with WATCHMAN.

**Methods:**

This was a retrospective study of 318 patients who underwent the WATCHMAN procedure in a tertiary referral center (June 2016-September 2020). We compared the outcomes of patients who were discharged on DOACs versus warfarin after the WATCHMAN procedure. The primary outcome was the composite of any bleeding, thromboembolism, or cardiovascular death through 7 ​days and 45 ​days after the procedure.

**Results:**

The final analysis included 301 patients, of whom 82.4% (248/301) were discharged on DOACs and 17.6% (53/301) were discharged on warfarin. The mean CHA_2_DS_2_-VASc and HAS-BLED scores were 4.9 ​± ​1.6 and 2.9 ​± ​0.9, respectively. The primary composite outcome was similar between the DOAC and warfarin groups through 7 ​days (3.2% vs 5.6%; adjusted odds ratio [OR], 0.65; 95% confidence interval [CI], 0.13-3.17; *P* ​= ​.59) and 45 days after procedure (10.1% vs 11.3%; adjusted OR, 1.18; 95% CI, 0.41-3.45; *P* ​= ​.76). Major bleeding (5.2% vs 9.5%; *P* ​= ​.34) and all-cause readmission (12.5% vs 16.9%; *P* ​= ​.85) at 45 ​days were comparable between the DOAC and warfarin groups. The overall incidence of device-related thrombus and significant peri-device flow at 45 ​days were low (<0.5%).

**Conclusions:**

In high-risk patients with atrial fibrillation, the primary composite outcome of any bleeding, thromboembolism, or cardiovascular death through 7 ​days and 45 ​days following WATCHMAN implantation was similar in patients receiving DOACs versus warfarin.

## Highlights

• The composite of bleeding, thromboembolism, or cardiovascular death was similar for DOAC vs warfarin after WATCHMAN.
• Overall, device-related thrombus and peri-device flow >5 ​mm were rare (<0.5%).
• DOACs are potentially safe and effective following WATCHMAN procedure.

## Central Illustration

**Figure:**
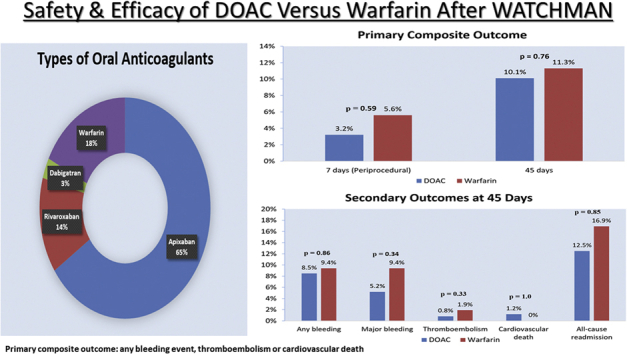

## Introduction

Atrial fibrillation (AF) is the most common cardiac arrhythmia in the United States. Percutaneous left atrial appendage closure (LAAC) is a nonpharmacologic option for the prevention of thromboembolism in AF patients with contraindications to long-term oral anticoagulation (OAC).^1^^,^^2^ In 2015, the WATCHMAN device was approved by the United States Food and Drug Administration for patients with nonvalvular AF who are at a higher risk of bleeding on OAC and is noninferior to warfarin in clinical trials.^3^^,^^4^ In the United States, patients undergoing percutaneous LAAC with WATCHMAN are typically maintained on OAC and aspirin for 45 ​days. If the peri-device leak is ​≤5 ​mm and device-related thrombus (DRT) is absent at 45 ​days on transesophageal echocardiography (TEE), patients are transitioned to dual-antiplatelet therapy (DAPT) for 6 ​months from the day of the procedure and then aspirin indefinitely. This practice is in accordance with the protocol of the pivotal WATCHMAN trials, where warfarin was used exclusively for 45 ​days after implantation.^3^^,^^4^ Due to warfarin’s narrow therapeutic window, the need for frequent international normalized ratio (INR) monitoring, and various food and drug interactions,^5^ direct oral anticoagulants (DOACs) may be a feasible alternative to warfarin for postprocedural anticoagulation following the WATCHMAN procedure.

DOACs were shown to be potentially safe and feasible for postprocedural OAC after WATCHMAN implantation in a retrospective, multicenter observational study.^6^ However, there is a paucity of data on the safety of DOACs following LAAC in high-risk patients with AF.^7^^,^^8^ In this retrospective single-center analysis, we sought to investigate the safety and efficacy of DOACs vs warfarin for postprocedural OAC following LAAC in AF patients at high risk for both bleeding and embolic events.

## Methods

### Patient selection

This was a retrospective analysis of consecutive patients who underwent LAAC using WATCHMAN at a tertiary referral center between June 2016 and September 2020.^9^ In our center, WATCHMAN is offered to AF patients at increased risk of stroke who have contraindications to long-term OAC in accordance with the current guidelines (class IIb recommendation).^2^ Institutional review board approval was obtained, and patient informed consent was waived for this study. Patients with unsuccessful LAAC and/or discharged without OAC following LAAC were excluded from the analysis.

### Procedure

Four operators performed LAAC in our hospital using WATCHMAN or WATCHMAN FLX. All procedures were performed using general anesthesia with fluoroscopy and TEE guidance. Anticoagulation with heparin was used in all patients to maintain an activated clotting time >250 seconds. WATCHMAN implantation was performed as previously described.^3^ A single Perclose was performed on all patients after LAAC to achieve hemostasis.

### Postprocedural care and follow-up

All patients were extubated in the catheterization laboratory after LAAC. Two hours after procedure, patients were ambulated to assess the integrity of the vascular access site. Patients without same-day procedure-related complications or immediate issues with the vascular access site were given the option of same-day discharge 3-4 ​hours after procedure or the following day.^10^ OAC was resumed or initiated 6-8 ​hours after procedure. Patients with successful LAAC were discharged on aspirin and OAC (DOACs or warfarin) for 45 ​days.

### Primary and secondary outcomes

The primary outcome was a composite of any bleeding event, thromboembolism, or cardiovascular death through 7 ​days (periprocedural) and 45 ​days following LAAC. The secondary outcomes were the individual components of the primary outcome, major bleeding events, vascular complications requiring endovascular intervention, all-cause readmission, and all-cause death through 7 ​days (periprocedural) and 45 ​days after procedure. Peri-device flow and DRT were assessed on 45-day TEE. Peri-device flow >5 ​mm was considered clinically significant.

Thromboembolism was defined as stroke, transient ischemic attack (TIA), or systemic embolism. Major bleeding was defined as bleeding into a critical organ, decrease in hemoglobin ≥2 ​g/dL from baseline, or transfusion ≥2 units of packed red blood cells.^11^ The remaining bleeding events were classified as minor bleeding.

### Statistical analysis

Continuous variables were presented as mean ​± ​standard deviation, and categorical variables were presented as counts and percentages. For continuous variables, the *t* test was performed to compare the means between DOAC and warfarin groups. The Pearson χ^2^ test was used to compare categorical variables between DOAC and warfarin groups; if any cells in a 2 ​× ​2 table contained an expected count less than 5, then the Fisher exact test was performed. Adjusted odds ratios (ORs) and 95% confidence intervals (CIs) for the primary and secondary outcomes were calculated using a multivariate logistic regression model. Covariates used for adjustment in the logistic regression model included age, sex, CHA_2_DS_2_-VASc score, HAS-BLED score, and baseline characteristics with statistically significant differences between the DOAC and warfarin groups (ie, abnormal renal function, OAC at LAAC referral, and concurrent antiplatelet upon discharge). A *P* value <.05 was considered statistically significant. Analyses were performed using IBM SPSS Statistics for Windows, version 28.0 (IBM Corp).

## Results

### Procedural outcomes

**Figure 1:**
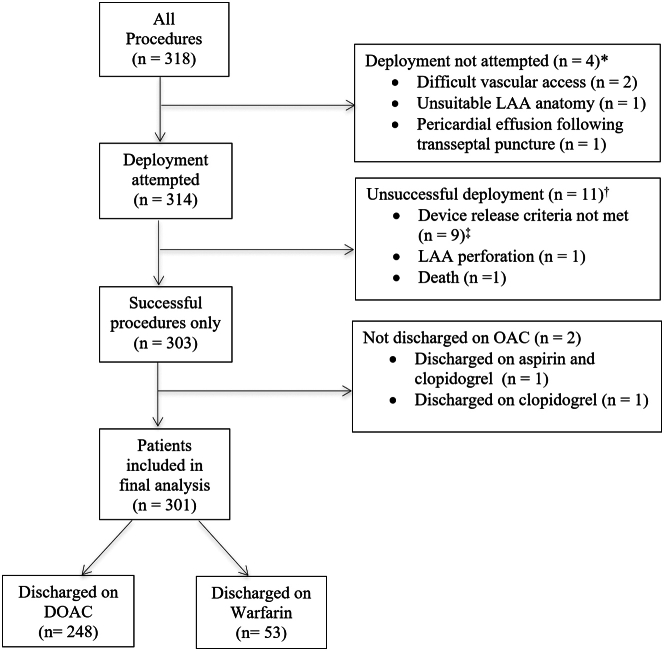
Flowchart of patients included in the final analysis. ∗Procedure aborted before device deployment. ^†^WATCHMAN deployed but unsuccessful. ^‡^All device release criteria, position, anchor, size, and seal (PASS), must be met for device release. LAA, left atrial appendage.

Between June 2016 and September 2020, 318 patients with nonvalvular AF underwent LAAC (Figure 1) with WATCHMAN (*n* ​= ​310) or WATCHMAN FLX (*n* ​= ​8). Preoperative imaging for LAA evaluation was performed in 67% (212/318) of patients using TEE (50.5%; 107/212) or cardiac computed tomography (49.5%; 105/212). Device deployment was not attempted in 4 patients (2 had difficult vascular access, 1 had unsuitable LAA anatomy, and 1 developed pericardial effusion following transseptal puncture). Two patients had persistent LAA thrombus despite OAC and underwent successful, uncomplicated WATCHMAN implantation with off-label use of transcatheter cerebral embolic protection.^12^

Device implantation was successful in 96.5% (303/314) of patients where device deployment was attempted. We excluded 11 aborted procedures from our analysis, which included 9 patients who did not meet device release criteria, 1 LAA perforation requiring emergent surgery, and 1 death related to cardiac perforation of the left atrium. Among the 303 patients who had successful device implantation, we further excluded 2 patients not discharged on OAC from our analysis, which included 1 patient who was discharged on aspirin and clopidogrel and 1 patient discharged on clopidogrel alone. Both patients had recent gastrointestinal (GI) bleeding and refused short-term OAC following WATCHMAN implantation. Thus, the final analysis included 301 patients (Figure 1).

### Patient characteristics

**Figure 2:**
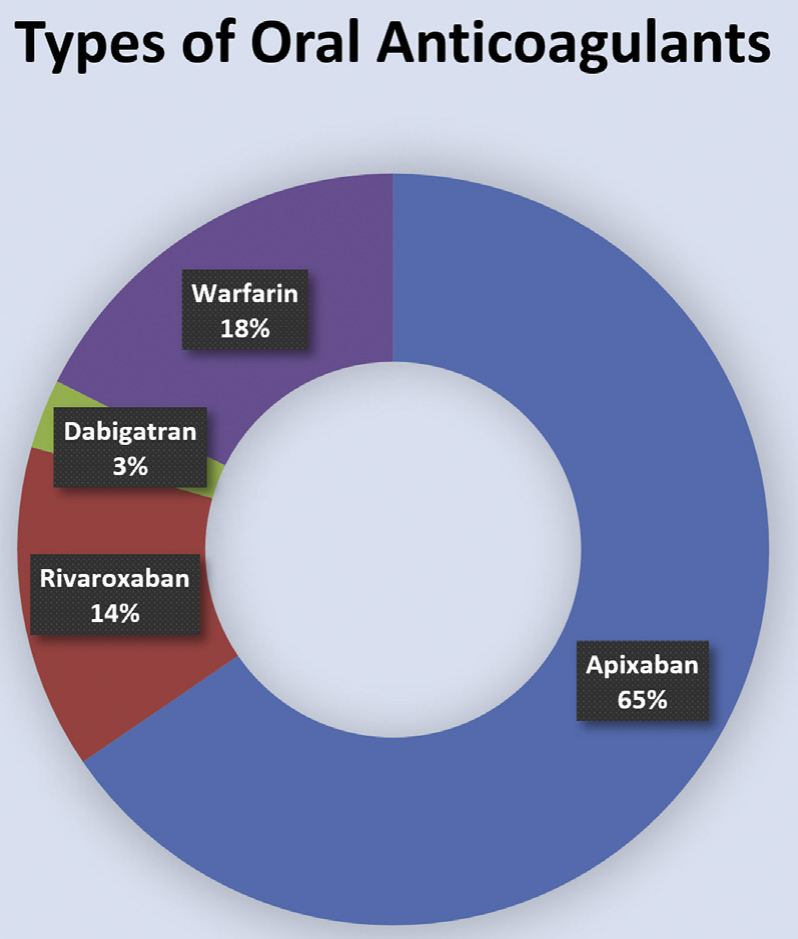
Types of oral anticoagulation after WATCHMAN procedure.

Among 301 patients included in the final analysis, 248 patients received DOACs and 53 patients received warfarin after WATCHMAN implantation. Within the DOAC group, 79.4% (197/248) received apixaban, 16.9% (42/248) received rivaroxaban, and 3.6% (9/248) received dabigatran (Figure 2). Antiplatelet therapy was also prescribed in 89.4% of patients (269/301) after implantation and included aspirin (260/269), clopidogrel (6/269), and ticagrelor (3/269).

Baseline characteristics are reported in Table 1. The mean CHA_2_DS_2_-VASc and HAS-BLED scores were 4.9 ​± ​1.6 and 2.9 ​± ​0.9, respectively. Overall, 50% (151/301) of patients were not on OAC upon referral to our center for LAAC, 80% (239/301) had a history of bleeding requiring hospitalization or transfusion, 10% (31/301) had a history of intracranial hemorrhage, and 27% (80/301) had high fall risks.

**Table 1:** Baseline and discharge characteristics

	All patients (*n* ​= ​301)	DOAC (*n* ​= ​248)	Warfarin (*n* ​= ​53)	*P*-value
Age, y	76.1 ​± ​8.4 (47, 94)	76.2 ​± ​8.2 (49, 94)	75.5 ​± ​9.3 (47, 92)	.53
Age ≥75	179 (59.5%)	151 (60.9%)	28 (52.8%)	.28
Female	125 (41.5%)	105 (42.3%)	20 (37.7%)	.54
CHA_2_DS_2_-VASc (categorical)				
1	1 (0.3%)	0 (0%)	1 (1.9%)	.18
2	6 (2.0%)	6 (2.4%)	0 (0%)	.59
3	54 (17.9%)	48 (19.4%)	6 (11.3%)	.17
4	72 (23.9%)	60 (24.2%)	12 (22.6%)	.81
5	72 (23.9%)	58 (23.4%)	14 (26.4%)	.64
6	39 (13.0%)	30 (12.1%)	9 (17.0%)	.34
7	36 (12.0%)	29 (11.7%)	7 (13.2%)	.76
8	16 (5.3%)	13 (5.2%)	3 (5.7%)	.51
9	5 (1.7%)	4 (1.6%)	1 (1.9%)	1.0
CHA_2_DS_2_-VASc (continuous)	4.9 ​± ​1.6 (1.0, 9.0)	4.9 ​± ​1.6 (2.0, 9.0)	5.2 ​± ​1.6 (1.0, 9.0)	.25
HAS-BLED (categorical)				
1	14 (4.7%)	11 (4.4%)	3 (5.7%)	.72
2	99 (32.9%)	89 (35.9%)	10 (18.9%)	.02
3	123 (40.9%)	100 (40.3%)	23 (43.4%)	.68
4	47 (15.6%)	38 (15.3%)	9 (17.0%)	.76
5	16 (5.3%)	9 (3.6%)	7 (13.2%)	.005
6	2 (0.7%)	1 (0.4%)	1 (1.9%)	.32
HAS-BLED (continuous)	2.9 ​± ​0.9 (1.0, 6.0)	2.8 ​± ​0.9 (1.0, 6.0)	3.2 ​± ​1.1 (1.0, 6.0)	.006
AF type				.92
Paroxysmal AF	142 (47.2%)	116 (46.8%)	26 (49.1%)	
Persistent AF	46 (15.3%)	38 (15.3%)	8 (15.1%)	
Permanent AF	112 (37.2%)	93 (37.5%)	19 (35.8%)	
Concurrent atrial flutter	27 (8.9%)	25 (10.1%)	2 (3.8%)	.18
Heart failure	143 (47.5%)	112 (45.2%)	31 (58.5%)	.08
Hypertension	288 (95.7%)	237 (95.6%)	51 (96.2%)	.83
Diabetes	111 (36.9%)	88 (35.5%)	23 (43.4%)	.28
Previous ischemic stroke/TIA	104 (34.6%)	81 (32.7%)	23 (43.4%)	.14
Prior MI	104 (34.6%)	86 (34.7%)	18 (34.0%)	.92
History of bleeding requiring hospitalization/transfusion	239 (79.4%)	196 (79.0%)	43 (81.1%)	.73
Intracranial bleeding	31 (10.3%)	27 (10.9%)	4 (7.5%)	.62
GI bleeding	139 (46.2%)	113 (45.6%)	26 (49.1%)	.64
High fall risk	80 (26.6%)	64 (25.8%)	16 (30.2%)	.51
Abnormal renal function^a^	40 (13.3%)	23 (9.3%)	17 (32.1%)	<.0001
Alcohol abuse	10 (3.3%)	9 (3.6%)	1 (1.9%)	1.0
On antiplatelet/NSAIDs	161 (53.5%)	133 (53.6%)	28 (52.8%)	.92
On OAC at referral for LAAC	150 (49.8%)	116 (46.8%)	34 (64.2%)	.02
Discharged on concurrent antiplatelet	269 (89.4%)	217 (87.5%)	52 (98.1%)	.02
Concurrent aspirin	260 (86.4%)	209 (84.3%)	51 (96.2%)	.03
Concurrent clopidogrel	6 (1.9%)	5 (2.0%)	1 (1.9%)	1.0
Concurrent ticagrelor	3 (0.9%)	3 (1.2%)	0 (0%)	1.0

Values are mean ​± ​SD (minimum, maximum) or *n* (%).

AF, atrial fibrillation; DOAC, direct oral anticoagulant; GI, gastrointestinal; LAAC, left atrial appendage closure; MI, myocardial infarction; NSAID, nonsteroidal anti-inflammatory drug; OAC, oral anticoagulation; TIA, transient ischemic attack.

a Abnormal renal function as defined in the HAS-BLED risk model, ie, recipient of kidney transplant or chronic dialysis or dialysis within 1 week prior to admission or serum creatinine ≥2.6 ​mg/dL.

At the time of referral to our center for LAAC, 50% (150/301) of patients were on OAC. Fewer patients were on OAC upon referral to our center for LAAC in the DOAC group (46.8%; 116/248; 112 DOACs and 4 warfarin) than in the warfarin group (64.2%; 34/53; 34 warfarin) (*P* ​= ​.02). Among the 150 patients who were on OAC upon referral, 63 patients (DOAC ​= ​40 and warfarin ​= ​23) had uninterrupted OAC (ie, OAC not held before the procedure) at the time of the WATCHMAN procedure.

Age, sex, CHA_2_DS_2_-VASc score, and most comorbidities were similar between the DOAC and warfarin groups, but the mean HAS-BLED score in the DOAC group was lower than that in the warfarin group (2.8 vs 3.2; *P* ​= ​.006). Abnormal renal function was less prevalent in the DOAC group than that in the warfarin group (9.3% vs 32.1%; *P* ​< ​.0001). Fewer patients in the DOAC group were discharged on concurrent antiplatelet agents than in the warfarin group (87.5% vs 98.1%; *P* ​= ​.02).

### Periprocedural outcomes

**Figure 3:**
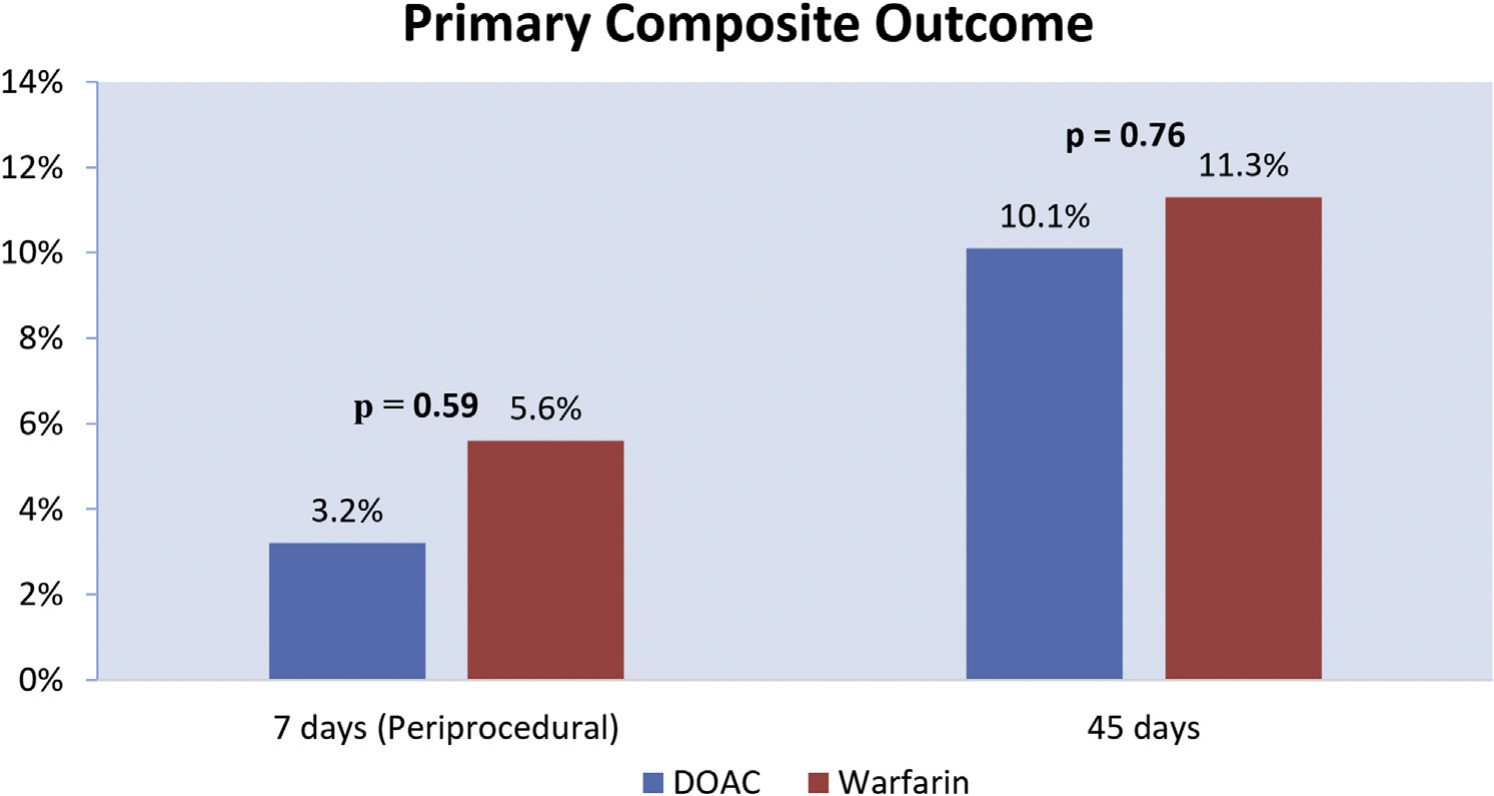
Primary composite outcome of any bleeding, thromboembolism, or cardiovascular death at 7 ​days and 45 ​days after WATCHMAN procedure. DOAC, direct oral anticoagulant.

There was no significant difference in the primary composite outcome between the DOAC and warfarin groups (3.2% vs 5.6%; adjusted OR, 0.65; 95% CI, 0.13-3.17; *P* ​= ​.59; Table 2, Figure 3). The endpoint of thromboembolism was similar between the DOAC and warfarin groups (0.8% vs 1.9%; adjusted OR, 0.24; 95% CI, 0.01-4.18; *P* ​= ​.33). Within the DOAC group, 1 patient had an ischemic stroke on OAC prior to discharge the day following LAAC and 1 patient had an ischemic stroke on OAC after discharge. In the warfarin group, 1 patient had a TIA 2 hours after LAAC before restarting warfarin.

**Table 2:** Periprocedural and 45-day outcomes

	DOAC (*n* ​= ​248)	Warfarin (*n* ​= ​53)	Unadjusted OR (95% CI)	Adjusted OR^e^ (95% CI)	*P* value
Primary outcome^a^					
Periprocedural^c^	8/248 (3.2%)	3/53 (5.6%)	0.56 (0.14, 2.20)	0.65 (0.13, 3.17)	.59
45 ​d^d^	25/248 (10.1%)	6/53 (11.3%)	0.88 (0.34, 2.23)	1.18 (0.41, 3.45)	.76
Any bleeding					
Periprocedural	6/248 (2.4%)	2/53 (3.8%)	0.63 (0.12, 3.20)	0.81 (0.13, 5.28)	.83
45 ​d	21/248 (8.5%)	5/53 (9.4%)	0.89 (0.32, 2.47)	1.11 (0.35, 3.47)	.86
Major bleeding					
Periprocedural	3/248 (1.2%)	2/53 (3.8%)	0.31 (0.05, 1.91)	0.49 (0.07, 3.47)	.47
45 ​d	13/248 (5.2%)	5/53 (9.4%)	0.53 (0.18, 1.56)	0.56 (0.17, 1.83)	.34
Thromboembolism^b^					
Periprocedural	2/248 (0.8%)	1/53 (1.9%)	0.42 (0.04, 4.75)	0.24 (0.01, 4.18)	.33
45 ​d	2/248 (0.8%)	1/53 (1.9%)	0.42 (0.04, 4.75)	0.24 (0.01, 4.18)	.33
Cardiovascular death					
Periprocedural	0/248 (0%)	0/53 (0%)	-	-	-
45 ​d	3/248 (1.2%)	0/53 (0%)	1.01 (0.99, 1.02)	-	1.0
All-cause death					
Periprocedural	0/248 (0%)	0/53 (0%)	-	-	-
45 ​d	3/248 (1.2%)	0/53 (0%)	1.01 (0.99, 1.02)	-	1.0
Vascular complications requiring surgery					
Periprocedural	1/248 (0.4%)	0/53 (0%)	1.00 (0.99, 1.01)	-	1.0
45 ​d	1/248 (0.4%)	0/53 (0%)	1.00 (0.99, 1.01)	-	1.0
All-cause readmission					
Periprocedural	8/248 (3.2%)	3/53 (5.7%)	0.56 (0.14, 2.7)	0.79 (0.17, 3.69)	.76
45 ​d	31/248 (12.5%)	9/53 (16.9%)	0.69 (0.31, 1.56)	0.92 (0.37, 2.26)	.85
DRT on 45-d TEE	1/223 (0.4%)	0/52 (0%)	1.00 (0.99, 1.01)	-	1.0
Peri-device flow >5 ​mm on 45-d TEE	0/223 (0%)	1/52 (1.9%)	0.98 (0.95, 1.02)	-	.18

Values are *n* (%).

CI, confidence interval; DOAC, direct oral anticoagulant; DRT, device-related thrombus; OR, odds ratio; TEE, transesophageal echocardiogram.

a Primary composite outcome: all bleeding events, thromboembolism, or cardiovascular death.

b Thromboembolism: ischemic stroke, transient ischemic attack, or systemic embolism.

c Periprocedural outcome: outcomes through 7 ​days after procedure.

d 45-Day outcome: outcomes through 45 ​days after procedure.

e ^|^Odds ratio adjusted for age, sex, CHA_2_DS_2_-VASc score, HAS-BLED score, abnormal renal function, anticoagulation at referral, and concurrent antiplatelet upon discharge using multivariate logistic regression.

There were no significant differences in any bleeding, major bleeding, vascular complication requiring endovascular interventions, and all-cause readmission. Periprocedural cardiovascular or all-cause death was not observed in any patient included in the final analysis.

### 45-Day outcomes

**Figure 4:**
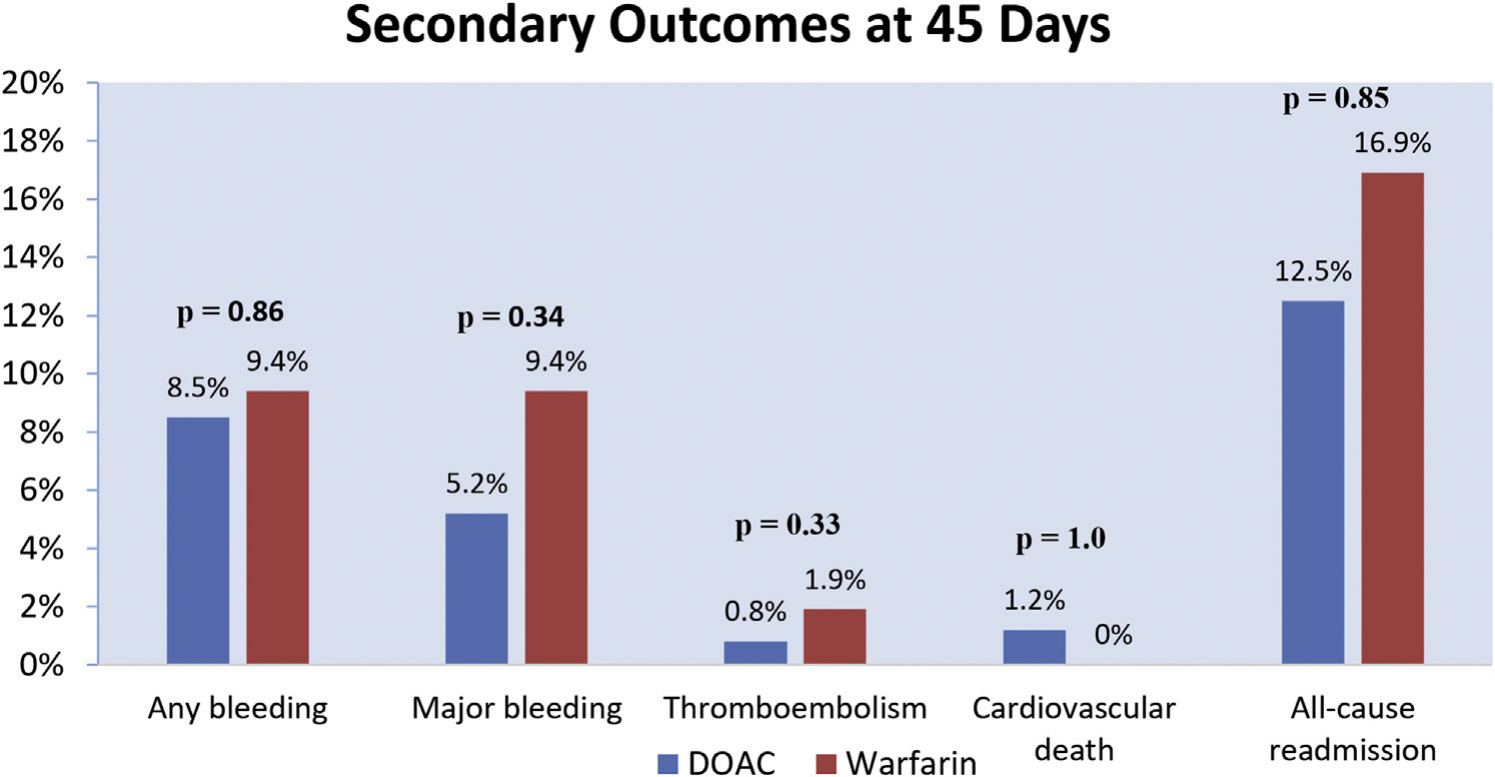
Key secondary outcomes at 45 ​days after WATCHMAN procedure. DOAC, direct oral anticoagulant.

All patients in our analysis (*n* ​= ​301) had complete follow-up through 45 ​days following LAAC. There was no significant difference in the primary composite outcome between the DOAC and warfarin groups (10.1% vs 11.3%; adjusted OR, 1.18; 95% CI, 0.41-3.45; *P* ​= ​.76; Table 2, Figure 3). The endpoint of thromboembolism was similar between the DOAC and warfarin groups (0.8% vs 1.9%; adjusted OR, 0.24; 95% CI, 0.01-4.18; *P* ​= ​.33; Figure 4), where all occurred periprocedurally (ie, 7 ​days after LAAC) with no additional events observed between 7 and 45 ​days. No significant differences were observed in any bleeding (8.5% vs 9.4%; adjusted OR, 1.11; 95% CI, 0.35-3.47; *P* ​= ​.86) or major bleeding (5.2% vs 9.4%; adjusted OR, 0.56; 95% CI, 0.17-1.83; *P* ​= ​.34) events between the DOAC and warfarin groups.

**Central Illustration:**
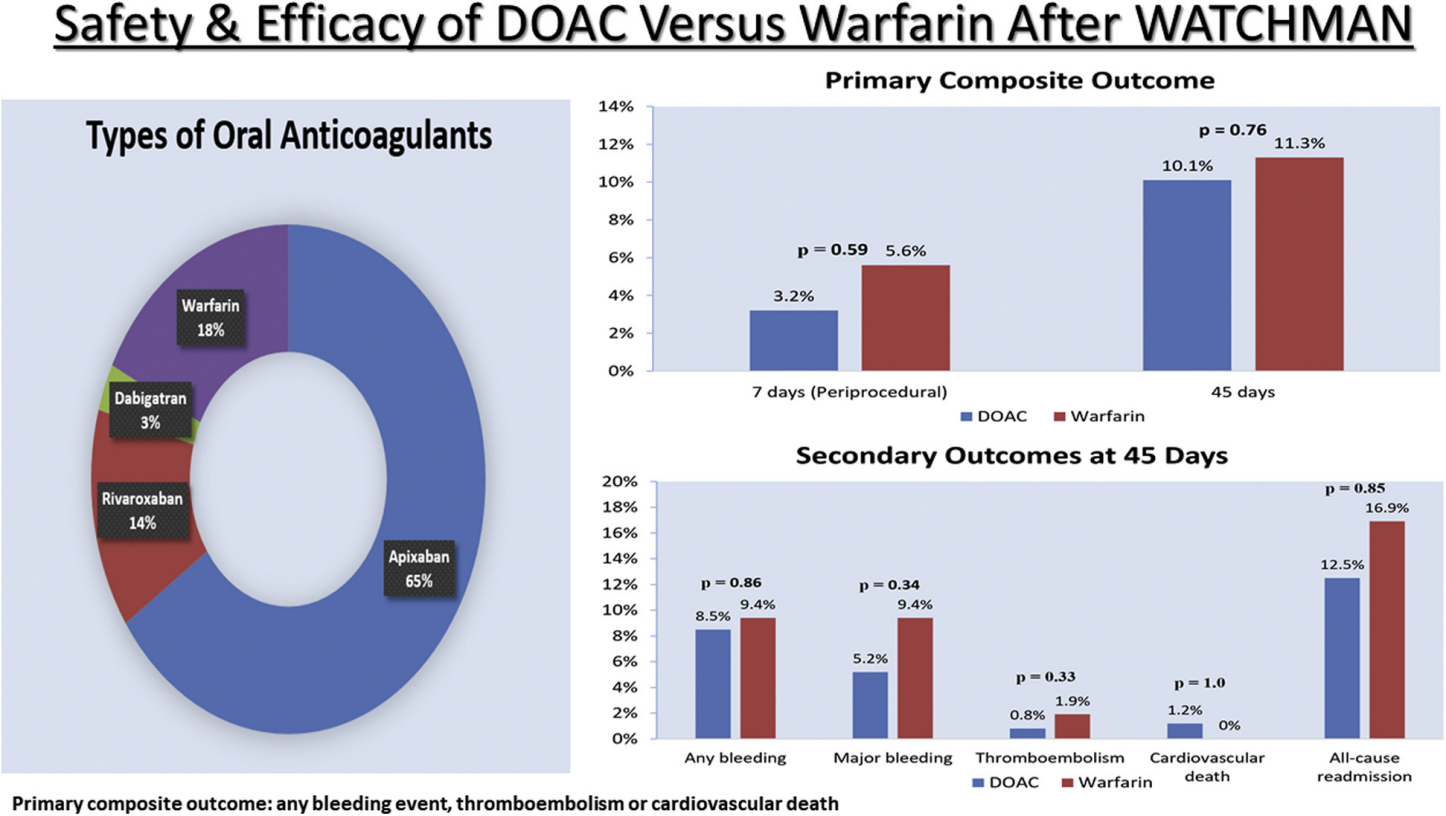
The safety and efficacy of direct oral anticoagulants versus warfarin after the WATCHMAN procedure.

Death occurred in 1.2% (3/248) of DOAC patients and 0% (0/53) of warfarin patients (*P* ​= ​1.0). All deaths were cardiovascular (1 ventricular fibrillation, 1 unwitnessed cardiac arrest, and 1 cardiogenic shock). Vascular complications requiring endovascular interventions occurred in 0.4% (1/248) of DOAC patients and 0% (0/53) of warfarin patients (*P* ​= ​1.0) at 7 ​days after procedure, with no events occurring between 7 ​days and 45 ​days. One patient was hospitalized for a femoral pseudoaneurysm and arteriovenous fistula requiring surgery.

The all-cause readmission rate was 12.5% (31/248) in the DOAC group and 16.9% (9/53) in the warfarin group (adjusted OR, 0.92; 95% CI, 0.37-2.26; *P* ​= ​.85). In the DOAC group, there were 41 readmissions in 31 patients. These included 16 major bleeding (15 GI bleeding and 1 groin hematoma with femoral pseudoaneurysm and arteriovenous fistula), 2 minor bleeding (both GI bleeding), 1 ischemic stroke, 14 cardiac-related readmissions (11 heart failure exacerbations and 3 rapid AF), and 8 noncardiac-related readmissions (1 pneumonia, 1 cellulitis, 1 GI infection, 1 acute kidney injury, 1 severe migraine, 1 noncardiogenic syncope, 1 mechanical fall, and 1 unclear reason). In the warfarin group, 4 patients had readmission for major bleeding (GI), 3 had cardiac-related readmissions for rapid AF, and 2 had noncardiac-related readmissions (1 pneumonia and 1 sepsis).

At 45 days after procedure, after excluding 3 patients from the DOAC group who died, 91.0% (223/245) from the DOAC group and 98.1% (52/53) from the warfarin group underwent surveillance TEE. There was no statistically significant difference in DRT (0.4% vs 0%; *P* ​= ​1.0) or peri-device flow >5 ​mm (0% vs 1.9%; *P* ​= ​.18). One patient from the DOAC group had DRT and 1 patient from the warfarin group had peri-device flow >5 ​mm on 45-day TEE (both patients were on OAC at 45 ​days). Both were maintained on OAC past the 45-day period.

During the 45-day period, after excluding 3 patients from the DOAC group who died, 95.9% (235/245) from the DOAC group and 100% (53/53) from the warfarin group remained on OAC at 45 ​days (*P* ​= ​.21). Among the 10 patients who discontinued OAC before 45 ​days, none had thromboembolism, DRT, or significant peri-device flow >5 ​mm at 45 ​days. The reasons for OAC discontinuation before 45 ​days (*n* ​= ​10) were 4 major GI bleeds, 1 minor bleed (epistaxis), 1 hospice care, 2 patient/physician preference, and 2 due to unclear reasons.

### 45-Day outcomes of DOAC subgroups

At 45 days after LAAC, there was no significant difference in the primary composite outcome in patients receiving apixaban vs warfarin (11.7% vs 11.3%; *P* ​= ​.94), apixaban vs nonapixaban DOACs (11.7% vs 3.9%; *P* ​= ​.12), or nonapixaban DOACs versus warfarin (3.9% vs 11.3%; *P* ​= ​.27) (Supplemental Tables). There was also no statistically significant difference in major bleeding at 45 ​days in patients receiving apixaban vs warfarin (5.6% vs 9.4%; *P* ​= ​.31), apixaban vs nonapixaban DOAC (5.6% vs 3.9%; *P* ​= ​1.0), or nonapixaban DOACs vs warfarin (3.9% vs 9.4%; *P* ​= ​.44).

## Discussion

Our study demonstrates the potential safety and efficacy of postprocedural OAC with DOACs compared with warfarin in high-risk patients with AF undergoing WATCHMAN implantation. The primary composite outcome (any bleeding event, thromboembolism, or cardiovascular death) and secondary outcomes at 7 ​days and 45 ​days after procedure were comparable between the 2 groups. Regardless of postprocedural anticoagulation strategy, the incidence of DRT and significant peri-device flow (>5 ​mm) at 45 ​days in our study were low (<0.5% overall) (Central Illustration).

Unlike the pivotal WATCHMAN trials (PROTECT-AF, PREVAIL)^3^^,^^4^ that recruited patients who were eligible for long-term OAC, our center offered WATCHMAN to nonvalvular AF patients with contraindications to long-term OAC due to elevated bleeding risks. In our study, 62.5% of patients had a HAS-BLED score ≥3, indicating high bleeding risks with OAC, compared with 20% in PROTECT-AF, 30% in PREVAIL, and 40% in EWOLUTION.^13^^,^^14^ Furthermore, half of our patients were not on OAC at the time of LAAC due to an elevated risk of bleeding, including nearly 80% with a history of bleeding requiring transfusion or hospitalization and 10% with a history of intracranial hemorrhage. The mean CHA_2_DS_2_-VASc score of patients who underwent WATCHMAN in our study is higher at 4.9 than that in the PROTECT-AF (CHA_2_DS_2_-VASc of 3.4)^3^, PREVAIL (CHA_2_DS_2_-VASc of 3.8)^4^, and EWOLUTION (CHA_2_DS_2_-VASc of 4.5)^13^ studies (Table 3), indicating higher risks for embolic events in our study population.

**Table 3:** Demographic and characteristics of WATCHMAN patients in previous studies compared with our study

	PROTECT-AF 2005-2008 (*n* ​= ​463, intervention group)	PREVAIL 2011-2013 (*n* ​= ​269, intervention group)	EWOLUTION 2013-2015 (*n* ​= ​1021)	Enomoto 2015-2016 (*n* ​= ​426)	Cohen 2015-2017 (*n* ​= ​90)	Fry 2016-2018 (*n* ​= ​163)∗	NCDR LAAO 2016-2018 (*n* ​= ​38,158)	Our study 2016-2020 (*n* ​= ​301)
Age, y	71.7 ​± ​8.8	74.0 ​± ​7.4	73.4 ​± ​8.9	DOAC: 76 ​± ​8.0 Warfarin: 75 ​± ​8.0	DOAC: 76.9 ​± ​8.7 Warfarin: 76.8 ​± ​7.5	80	76.1 ​± ​8.1	76.1 ​± ​8.4
Female	137 (29.6%)	87 (32.3%)	411 (40.1%)	DOAC: 80 (37%) Warfarin: 65 (31%)	DOAC: 17 (32.7%) Warfarin: 19 (42.4%)	69 (42.3%)	15,672 (41.1%)	125 (41.5%)
CHA_2_DS_2_-VASc score	3.4 ​± ​1.5	3.8 ​± ​1.2	4.5 ​± ​1.6	DOAC: 3.8 ​± ​1.4 Warfarin: 4.1 ​± ​1.4	DOAC: 4.7 ​± ​1.5 Warfarin: 4.7 ​± ​1.5	4.9 ​± ​1.5	4.6 ​± ​1.5	4.9 ​± ​1.6
HAS-BLED score	N/A	N/A	2.3 ​± ​1.2	DOAC: 2.4 ​± ​1.0 Warfarin: 2.7 ​± ​0.9	DOAC: 3.5 ​± ​1.0 Warfarin: 3.5 ​± ​0.8	2.6 ​± ​0.9	3.0 ​± ​1.1	2.9 ​± ​0.9
History of ischemic stroke/TIA	82 (17.7%)	74 (27.5%)	312 (30.5%)	N/A	DOAC: 23 (44.2%) Warfarin: 12 (35.6%)	37 (22.6%)	11,389 (29.9%)	104 (34.6%)
History of bleeding requiring hospitalization/transfusion	N/A	N/A	316 (31.2%)	N/A	N/A	N/A	26,466 (69.4%)	239 (79.4%)
History of intracranial hemorrhage	N/A	N/A	152 (15.0%)	N/A	DOAC: 14 (26.9%) Warfarin: 10 (22.2%)	9 (5.5%)	4550 (11.9%)	31 (10.3%)

Values are mean ​± ​SD or *n* (%) for all studies, except for the study by ∗Fry et al, where age is provided in median.

DOAC, direct oral anticoagulant; TIA, transient ischemic attack.

The National Cardiovascular Data Registry LAA Occlusion registry^15^ reported a mean CHA_2_DS_2_-VASc score of 4.6 and a HAS-BLED score of 3.0, and 70% of patients had a history of clinically relevant bleeding. Similar to the National Cardiovascular Data Registry LAA Occlusion registry, our patient population was older, was sicker, and had an elevated risk of stroke and bleeding compared with earlier studies^3^^,^^4^^,^^13^ (Table 3). Thus, the findings of our study should be applicable to the patients undergoing LAAC in the current real-world clinical setting.

In randomized trials, DOACs are shown to be superior (apixaban and dabigatran 150 ​mg twice daily) or noninferior (rivaroxaban, edoxaban, and dabigatran 110 ​mg twice daily) to warfarin for the prevention of embolic events in AF.^3^^,^^4^ In addition, DOACs are associated with lower (apixaban, edoxaban, and dabigatran 110 ​mg twice daily) or similar rates (rivaroxaban and dabigatran 150 ​mg twice daily) of major bleeding compared with warfarin in AF patients.^1^^,^^2^ As a class, DOACs cause significantly less intracranial hemorrhage than warfarin. Our study population comprised a group of AF patients with high bleeding risks compared with the early WATCHMAN trials.^3^^,^^4^^,^^13^ As a result, the majority of patients (82.4%) who underwent LAAC at our center received DOACs after implantation.

A retrospective observational study by Enomoto et al^6^ (Table 3) compared DOAC (*n* ​= ​214) vs warfarin (*n* ​= ​212) for postprocedural OAC after WATCHMAN implantation and found no significant differences in bleeding and embolic events between the DOAC and warfarin groups. Any bleeding and major bleeding events occurred in 2.6% (11/426) and 1.6% (7/426) of patients at the time of follow-up TEE (performed between 6 ​weeks and 4 ​months after LAAC), compared with 8.6% (26/301) and 5.9% (18/301) of patients in our study at 45 ​days. In our study, the higher overall bleeding rates are due to an elevated baseline bleeding risk of our study population as compared with the study by Enomoto et al^6^ (our study vs Enomoto et al: mean HAS-BLED score of 2.9 vs 2.4-2.7; HAS-BLED score ≥3 in 62.5% vs 52.5%). Despite the higher CHA_2_DS_2_-VASc score in our study (our study vs Enomoto et al: score of 4.9 vs 3.8-4.1), the overall rates of thromboembolism in both studies were low (our study vs Enomoto et al: 0.9% vs 0.5%) during the postprocedural period due to the prevention of LAA thrombus formation with the use of WATCHMAN and OAC before device endothelialization.

In line with the findings of our study, smaller retrospective studies investigating high-risk AF patients (Cohen et al^7^: *n* ​= ​90, CHA_2_DS_2_-VASc score = 4.7, HAS-BLED score = 3.5; Fry et al^8^: *n* ​= ​158, CHA_2_DS_2_-VASc score = 4.9, HAS-BLED score = 2.6) (Table 3) also found no significant differences in bleeding and embolic events between the DOAC and warfarin groups following WATCHMAN implantation. Given the similar findings in these prior studies with our analysis,^6^, ^7^, ^8^ the collective data further support the potential safety and efficacy of DOACs following LAAC with WATCHMAN.

The 2019 American College of Cardiology/American Heart Association/Heart Rhythm Society guidelines recommend DOACs over warfarin for the prevention of embolic events in AF.^2^ DOACs do not require INR monitoring or dosage adjustment according to the INR. Furthermore, DOACs have fewer drug or food interactions compared with warfarin. As a result, patients taking DOACs have greater treatment adherence^16^ and patient satisfaction than those taking warfarin,^16^^,^^17^ with comparable quality of life metrics.^18^ Thus, post-LAAC anticoagulation with DOACs has the potential to enhance patient satisfaction and compliance without an increase in adverse outcomes. Additionally, apixaban, edoxaban, and dabigatran (110 ​mg twice daily) have the potential to lower bleeding risks.^1^^,^^2^

In the Amulet investigation device exemption trial, the Amplatzer Amulet occluder was noninferior compared with WATCHMAN for the primary safety outcome (composite of procedure-related complications, all-cause death, or major bleeding at 12 ​months) and the effectiveness outcome (composite of ischemic stroke or systemic embolism at 18 ​months).^19^ It is worth noting that the majority of Amulet patients (75.7%) received DAPT without OAC after the procedure. As such, Amulet is a promising option for AF patients with absolute contraindications to OAC that preclude them from receiving WATCHMAN which typically requires 45-day postprocedural OAC.^19^

Several important randomized trials investigating LAAC are currently underway. To inform physicians whether percutaneous LAAC is a reasonable alternative to DOAC in AF patients, 2 ongoing randomized trials are investigating LAAC with WATCHMAN-FLX (CHAMPION-AF trial)^20^ and Amulet (CATALYST trial)^21^ vs DOACs in AF patients who are eligible for long-term OAC. Additionally, the ASAP-TOO trial will assess the safety and efficacy of WATCHMAN in AF patients deemed ineligible for OAC by comparing WATCHMAN implantation using short-term DAPT vs single antiplatelet therapy or no therapy.^22^

## Limitations

Our study has some important limitations. First, this was a retrospective cohort study with inherent risks for selection bias, confounding bias (measured and unmeasured), and the inability to attribute causation. Potential selection bias involves the higher likelihood of patients with abnormal renal function receiving warfarin as postprocedural OAC compared with DOACs. There were some statistically significant differences in baseline characteristics between the 2 groups including HAS-BLED score, renal dysfunction, OAC at LAAC referral, and concurrent antiplatelet on discharge. However, a multivariate logistic regression model was used to adjust for these covariates. Second, approximately 80% of patients from the DOAC group received apixaban; as such, the conclusions of our primary analysis should be interpreted with caution for patients receiving nonapixaban DOACs. Third, given the modest sample size and number of adverse clinical events, our study is underpowered to detect statistically significant differences in the clinical outcomes. Fourth, we did not investigate long-term clinical outcomes following LAAC between groups. Given that OAC was discontinued in nearly all patients after the 45-day TEE, clinically relevant outcomes that occurred after OAC discontinuation were less likely attributable to OAC.

## Conclusions

In high-risk patients with AF, the primary composite outcome of any bleeding, thromboembolism, or cardiovascular death through 7 ​days and 45 ​days following WATCHMAN implantation was similar in patients receiving DOACs vs warfarin. The individual components of the primary outcome, major bleeding events, vascular complications requiring endovascular interventions, all-cause death, DRT, and peri-device flow >5 ​mm (at 45 ​days) between the 2 groups were comparable. DOACs appear to be safe with similar efficacy compared with warfarin for postprocedural anticoagulation following LAAC with WATCHMAN. The administration of DOACs has the potential to improve treatment adherence and patient satisfaction. Prospective clinical trials are warranted to validate the findings of our study.

## Declaration of competing interest

Dr. Jeremiah P. Depta discloses the following relationships: Consultant/Advisory Board: Edwards Lifesciences, Boston Scientific, and WL Gore & Associates. Dr Deepak L. Bhatt discloses the following relationships: Advisory Board: Bayer, Boehringer Ingelheim, Cardax, CellProthera, Cereno Scientific, Elsevier Practice Update Cardiology, Janssen, Level Ex, Medscape Cardiology, MyoKardia, NirvaMed, Novo Nordisk, PhaseBio, PLx Pharma, Regado Biosciences, and Stasys; Board of Directors: Boston VA Research Institute, DRS.LINQ (stock options), Society of Cardiovascular Patient Care, and TobeSoft; Chair: Inaugural Chair, American Heart Association Quality Oversight Committee; Data Monitoring Committees: Acesion Pharma, Assistance Publique-Hôpitaux de Paris, Baim Institute for Clinical Research (formerly Harvard Clinical Research Institute, for the PORTICO trial, funded by St. Jude Medical, now Abbott), Boston Scientific (Chair, PEITHO trial), Cleveland Clinic (including for the ExCEED trial, funded by Edwards), Contego Medical (Chair, PERFORMANCE 2), Duke Clinical Research Institute, Mayo Clinic, Mount Sinai School of Medicine (for the ENVISAGE trial, funded by Daiichi Sankyo; for the ABILITY-DM trial, funded by Concept Medical), Novartis, Population Health Research Institute; Rutgers University (for the NIH-funded MINT Trial); Honoraria: American College of Cardiology (Senior Associate Editor, Clinical Trials and News, ACC.org; Chair, ACC Accreditation Oversight Committee), Arnold and Porter law firm (work related to Sanofi/Bristol-Myers Squibb clopidogrel litigation), Baim Institute for Clinical Research (formerly Harvard Clinical Research Institute; RE-DUAL PCI clinical trial steering committee funded by Boehringer Ingelheim; AEGIS-II executive committee funded by CSL Behring), Belvoir Publications (Editor in Chief, Harvard Heart Letter), Canadian Medical and Surgical Knowledge Translation Research Group (clinical trial steering committees), Cowen and Company, Duke Clinical Research Institute (clinical trial steering committees, including for the PRONOUNCE trial, funded by Ferring Pharmaceuticals), HMP Global (Editor in Chief, Journal of Invasive Cardiology), Journal of the American College of Cardiology (Guest Editor; Associate Editor), K2P (Co-Chair, interdisciplinary curriculum), Level Ex, Medtelligence/ReachMD (CME steering committees), MJH Life Sciences, Piper Sandler, Population Health Research Institute (for the COMPASS operations committee, publications committee, steering committee, and USA national co-leader, funded by Bayer), Slack Publications (Chief Medical Editor, Cardiology Today’s Intervention), Society of Cardiovascular Patient Care (Secretary/Treasurer), WebMD (CME steering committees); other: Clinical Cardiology (Deputy Editor), NCDR-ACTION Registry Steering Committee (Chair), VA CART Research and Publications Committee (Chair); Research Funding: Abbott, Afimmune, Aker Biomarine, Amarin, Amgen, AstraZeneca, Bayer, Beren, Boehringer Ingelheim, Bristol Myers Squibb, Cardax, CellProthera, Cereno Scientific, Chiesi, CSL Behring, Eisai, Ethicon, Faraday Pharmaceuticals, Ferring Pharmaceuticals, Forest Laboratories, Fractyl, Garmin, HLS Therapeutics, Idorsia, Ironwood, Ischemix, Janssen, Javelin, Lexicon, Lilly, Medtronic, Moderna, MyoKardia, NirvaMed, Novartis, Novo Nordisk, Owkin, Pfizer, PhaseBio, PLx Pharma, Recardio, Regeneron, Reid Hoffman Foundation, Roche, Sanofi, Stasys, Synaptic, The Medicines Company, and 89Bio; Royalties: Elsevier (Editor, Cardiovascular Intervention: A Companion to Braunwald’s Heart Disease); Site Co-Investigator: Abbott, Biotronik, Boston Scientific, CSI, St. Jude Medical (now Abbott), Philips, and Svelte; Trustee: American College of Cardiology; and unfunded research: FlowCo, Merck, and Takeda. The other authors have no relevant disclosures.
