# Supplementary material for: Safety and Efficacy of Direct Oral Anticoagulants Versus Warfarin Following WATCHMAN in High-Risk Patients
Source: J Soc Cardiovasc Angiogr Interv. 2022 Apr 20;1(3):100042. doi: 10.1016/j.jscai.2022.100042 (PMC11307383; doi:10.1016/j.jscai.2022.100042)
Supplement: Supplemental Tables S1–S3 [file mmc1.docx]

**Supplementary table 1. 45-day outcomes (apixaban versus warfarin)**

|  | **Apixaban**  **(n=197)** | **Warfarin**  **(n=53)** | **p-value** |
| --- | --- | --- | --- |
| **Primary composite outcome***  45 days | 23/197 (11.7%) | 6/53 (11.3%) | 0.94 |
| **Any bleeding event**  45 days | 19/197 (9.6%) | 5/53 (9.4%) | 0.96 |
| **Major bleeding event**  45 days | 11/197 (5.6%) | 5/53 (9.4%) | 0.31 |
| **Thromboembolism**  45 days | 2/197 (1.0%) | 1/53 (1.0%) | 0.51 |
| **Cardiovascular death**  45 days | 3/248 (1.2%) | 0/53 (0%) | 1.0 |

Values are n (%). *Primary composite outcome: all bleeding events, stroke/TIA/systemic embolism or cardiovascular death.

**Supplementary table 2. 45-day outcomes (apixaban versus non-apixaban DOAC)**

|  | **Apixaban**  **(n=197)** | **Non-Apixaban DOAC (n=51)** | **p-value** |
| --- | --- | --- | --- |
| **Primary composite outcome***  45 days | 23/197 (11.7%) | 2/51 (3.9%) | 0.12 |
| **Any bleeding event**  45 days | 19/197 (9.6%) | 2/51 (3.9%) | 0.26 |
| **Major bleeding event**  45 days | 11/197 (5.6%) | 2/51 (3.9%) | 1.0 |
| **Thromboembolism**  45 days | 2/197 (1.0%) | 0/51 (0%) | 1.0 |
| **Cardiovascular death**  45 days | 3/248 (1.2%) | 0/51 (0%) | 1.0 |

Values are n (%). *Primary composite outcome: all bleeding events, stroke/TIA/systemic embolism or cardiovascular death.

**Supplementary table 3. 45-day outcomes (non-apixaban DOAC versus warfarin)**

|  | **Non-apixaban DOAC (n=51)** | **Warfarin**  **(n=53)** | **p-value** |
| --- | --- | --- | --- |
| **Primary composite outcome***  45 days | 2/51 (3.9%) | 6/53 (11.3%) | 0.27 |
| **Any bleeding event**  45 days | 2/51 (3.9%) | 5/53 (9.4%) | 0.44 |
| **Major bleeding event**  45 days | 2/51 (3.9%) | 5/53 (9.4%) | 0.44 |
| **Thromboembolism**  45 days | 0/51 (0%) | 1/53 (1.0%) | 1.0 |
| **Cardiovascular death**  45 days | 0/51 (0%) | 0/53 (0%) |  |

Values are n (%). *Primary composite outcome: all bleeding events, stroke/TIA/systemic embolism or cardiovascular death.
